# Nitric Oxide, Oxidative Stress and Endothelial Dysfunction in Migraine: Recent Advances and Molecular Mechanisms

**DOI:** 10.3390/ijms27093710

**Published:** 2026-04-22

**Authors:** Alexandra Ina Bulboacă, Alexandru Gerdanovics, Bogdan-Andrei Borlea, Ioana Cristina Stănescu, Gabriela Bombonica Dogaru, Cristina Ariadna Nicula, Camelia Manuela Mîrza, Adriana Elena Bulboacă

**Affiliations:** 1 Neurology Department, Schön Klinik, Parzivalpl. Str. 4, 80804 München, Germany; 2 Pathophysiology Department, “Iuliu Hațieganu” University of Medicine and Pharmacy, Victor Babeș Street, No. 2-4, 400012 Cluj-Napoca, Romania; 3 Clinical Rehabilitation Hospital Cluj-Napoca, Viilor Street, No. 46-50, 400066 Cluj-Napoca, Romania; 4 Faculty of Medicine, “Iuliu Hațieganu” University of Medicine and Pharmacy, Victor Babeș Street, No. 8, 400012 Cluj-Napoca, Romania; borlea.bogdan.andrei@elearn.umfcluj.ro; 5 Neurology Department, “Iuliu Hațieganu” University of Medicine and Pharmacy, Victor Babeș Street, No. 43, 400012 Cluj-Napoca, Romania; 6 Department of Balneophysiokinetotherapy and Medical Recovery, Faculty of Nursing and Health Sciences, “Iuliu Hațieganu” University of Medicine and Pharmacy, Victor Babeş Street, No. 8, 400347 Cluj-Napoca, Romania; 7 Medical Specialties, Department 1—Maxilo-Facial Surgery, Faculty of Dental Medicine, “Iuliu Haţieganu” University of Medicine and Pharmacy, Clinicilor Street, No. 3-5, 400006 Cluj-Napoca, Romania

**Keywords:** migraine, oxidative stress, nitric oxide, endothelial dysfunction, neuroinflammation

## Abstract

Migraine is a highly prevalent and disabling neurovascular disorder that represents a major global health burden due to its significant impact on quality of life and socioeconomic costs. Increasing evidence suggests that migraine pathophysiology involves complex interactions between neuronal hyperexcitability, vascular dysregulation, oxidative stress, and neuroinflammatory processes. Oxidative and nitrosative stress are increasingly recognized as key contributors to migraine mechanisms, influencing mitochondrial dysfunction, cortical spreading depression, and trigeminovascular activation. Nitric oxide plays a central role in these processes by regulating vascular tone, nociceptive signaling, and neurogenic inflammation through downstream pathways such as the soluble guanylate cyclase–cyclic guanosine monophosphate (NO–sGC–cGMP) signaling cascade. Dysregulation of nitric oxide signaling and increased oxidative stress may contribute to endothelial dysfunction and impaired cerebrovascular regulation observed in migraine patients. In addition, accumulating evidence highlights the role of neuroinflammatory mechanisms, including microglial activation and cytokine-mediated signaling, which may amplify nociceptive transmission within trigeminal pathways. Migraine is increasingly recognized as a systemic disorder associated with several comorbid conditions, including Parkinson’s disease, fibromyalgia, and autoimmune diseases such as Sjögren’s syndrome. This review summarizes recent advances regarding the interactions between oxidative stress, nitric oxide signaling, endothelial dysfunction, and neuroinflammation in migraine and discusses their potential therapeutic implications.

## 1. Introduction

Migraine is a highly prevalent and disabling neurovascular disorder characterized by recurrent attacks of moderate to severe headache often accompanied by nausea, photophobia, phonophobia, and sensory disturbances [1,2,3]. Affecting approximately 10–15% of the global population, migraine represents a major public health concern due to its significant impact on quality of life and socioeconomic burden [2,3]. Migraine prevalence shows a strong sex disparity, affecting approximately 18% of women and 6% of men, with a peak incidence between 18 and 44 years [2,3]. According to the International Classification of Headache Disorders, migraine belongs to the group of primary headache disorders, meaning that it is not caused by a structural lesion of the nervous system [4].

Current evidence suggests that migraine arises from a complex interaction between neuronal hyperexcitability, vascular dysfunction, genetic predisposition, and environmental triggers. Among the mechanisms involved, cortical spreading depression (CSD), trigeminovascular system activation, and the release of vasoactive neuropeptides such as calcitonin gene-related peptide (CGRP) play central roles in the initiation and propagation of migraine attacks [5]. CSD is characterized by a propagating wave of neuronal and glial depolarization followed by transient suppression of cortical activity, subsequently leading to significant alterations in neuronal functions and cerebral vessel dynamics [6,7,8,9]. Activation of the trigeminovascular system leads to the release of vasoactive mediators such as nitric oxide (NO) and CGRP, which cause pain and blood vessel dilation [9]. Additionally, specific mitochondrial DNA variations and polymorphisms in this region have been associated with migraine [7].

In recent years, increasing attention has been directed toward the contribution of oxidative and nitro-oxidative stress to migraine pathophysiology. Several studies have demonstrated that reactive oxygen and nitrogen species (ROS and RNS) can influence neuronal excitability, mitochondrial function, and neurovascular signaling, thereby promoting mechanisms that facilitate migraine attacks [10].

NO is considered one of the key mediators linking oxidative stress, endothelial dysfunction, and neurogenic inflammation in migraine. NO is generated from L-arginine by nitric oxide synthase (NOS) isoforms and participates in vascular tone regulation and nociceptive signaling, while excessive production may contribute to trigeminovascular activation and the release of CGRP [9]. Experimental models have shown that NO donors can trigger migraine-like attacks, highlighting the importance of this molecule in migraine pathogenesis [9,10].

Beyond its direct neuronal effects, NO also plays an important role in endothelial function and cerebrovascular regulation. Alterations in endothelial signaling pathways and redox balance may contribute to vascular dysregulation and to the increased risk of cerebrovascular events observed in migraine patients, particularly in those manifesting migraine with aura [11].

In this context, growing evidence suggests that oxidative stress, nitrosative stress, and inflammation represent interconnected processes that contribute to migraine pathophysiology. Understanding these molecular mechanisms may provide new insights into disease development and may support the identification of novel therapeutic targets. Therefore, this review aims to summarize recent advances in the understanding of nitric oxide signaling, oxidative stress, and endothelial dysfunction in migraine, highlighting the molecular mechanisms involved and their potential therapeutic implications.

**Figure 1:**
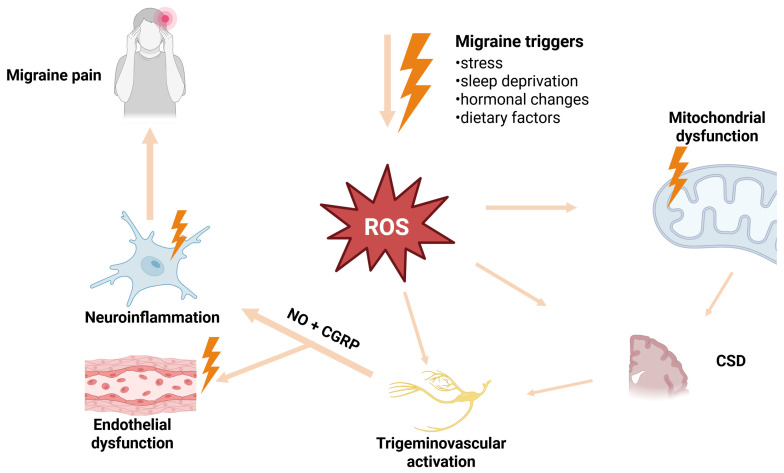
Oxidative stress–driven mechanisms involved in migraine pathophysiology. Migraine triggers such as stress, sleep deprivation, hormonal changes, and dietary factors may increase the production of ROS, promoting mitochondrial dysfunction and CSD. These processes contribute to trigeminovascular activation and the release of vasoactive mediators, including NO and CGRP, leading to endothelial dysfunction, neuroinflammation, and ultimately migraine pain. ROS—reactive oxygen species; CSD—cortical spreading depression; NO—nitric oxide; CGRP—calcitonin gene-related peptide.

The complex interactions between oxidative stress, mitochondrial dysfunction, cortical spreading depression, trigeminovascular activation, and endothelial dysfunction in migraine are summarized in Figure 1.

In this review, we primarily focus on molecular and biochemical mechanisms related to oxidative stress, nitric oxide signaling, endothelial dysfunction, and neuroinflammation in migraine, as well as on therapeutic approaches that are mechanistically linked to these pathways.

## 2. Oxidative Stress in Migraine

Oxidative stress has increasingly been implicated in the pathophysiology of migraine, contributing to neuronal dysfunction, mitochondrial impairment, and neurovascular dysregulation. ROS and RNS are generated during normal cellular metabolism and participate in physiological signaling processes; however, excessive production or insufficient antioxidant defenses may disrupt cellular homeostasis and promote tissue damage [10]. In the nervous system, oxidative stress may influence neuronal excitability and mitochondrial energy metabolism, mechanisms closely related to migraine pathogenesis [5,6,7,8,9,10]. Experimental and clinical studies suggest that alterations in redox balance may facilitate CSD, promote the release of vasoactive mediators, and contribute to neurogenic inflammation during migraine attacks [6,7,8,9,10]. Therefore, understanding the sources and molecular consequences of oxidative stress may provide important insights into the mechanisms underlying migraine and may highlight potential therapeutic targets.

### 2.1. Sources of Reactive Oxygen and Nitrogen Species Relevant to Migraine

Oxidative stress results from an imbalance between the production of ROS and RNS and the capacity of antioxidant defense systems to neutralize these reactive molecules [12]. Under physiological conditions, ROS are generated as by-products of aerobic cellular metabolism and participate in intracellular signaling and host defense mechanisms. However, excessive ROS accumulation can promote oxidative damage to lipids, proteins, and nucleic acids, thereby contributing to cellular dysfunction [12,13].

Mitochondria represent the major intracellular source of ROS, primarily through electron leakage from the mitochondrial respiratory chain during oxidative phosphorylation [14]. In addition to mitochondria, several enzymatic systems contribute to ROS generation, including NADPH oxidases (NOXs), which are important sources of ROS in vascular and neuronal tissues and may contribute to redox imbalance and neurovascular dysfunction in migraine, as well as xanthine oxidase, nitric oxide synthases, and various peroxisomal enzymes [15]. Oxidative species may also arise from exogenous sources such as ionizing radiation, ultraviolet exposure, or xenobiotic metabolism [13].

RNS constitute another important group of oxidant molecules involved in redox signaling. NO, the principal RNS, is produced from L-arginine by NOS isoforms, including neuronal (nNOS), endothelial (eNOS), and inducible (iNOS) forms [16,17,18]. Dysregulation of these enzymatic pathways may disturb cellular redox balance and contribute to oxidative and nitrosative stress in neurological disorders.

### 2.2. Mitochondrial Dysfunction and Energy Deficit in Migraine

Accumulating evidence suggests that mitochondrial dysfunction and impaired energy metabolism play an important role in migraine susceptibility. Migraine has been proposed to involve a cerebral energy deficit, which may arise from increased metabolic demand associated with neuronal hyperexcitability or from reduced energy production due to mitochondrial dysfunction [19,20].

Magnetic resonance spectroscopy (MRS) studies have provided important insights into brain energy metabolism in migraine patients. Investigations using phosphorus magnetic resonance spectroscopy (^31^P-MRS) have demonstrated abnormalities in mitochondrial oxidative phosphorylation both during migraine attacks and in the interictal period, suggesting persistent alterations in cerebral energy metabolism [19]. Similar metabolic changes have also been observed in peripheral tissues such as skeletal muscle and platelets, supporting the hypothesis that mitochondrial dysfunction in migraine may represent a systemic rather than a purely cerebral phenomenon [19].

Further evidence comes from biochemical studies showing reduced activity of several mitochondrial enzymes involved in oxidative metabolism, including monoamine oxidase, succinate dehydrogenase, NADH dehydrogenase, cyclooxygenase, and citrate synthase in platelets of migraine patients [19]. Because mitochondrial DNA is particularly susceptible to oxidative damage, oxidative stress may further impair mitochondrial function and contribute to the energetic vulnerability observed in migraine [20].

### 2.3. Cortical Spreading Depression and Oxidative Damage

CSD represents a fundamental neurophysiological mechanism implicated in migraine pathogenesis, particularly in migraine with aura. CSD consists of a slowly propagating wave of neuronal and glial depolarization that travels across the cerebral cortex at a rate of approximately 3–5 mm/min, followed by a transient suppression of neuronal activity. This phenomenon is associated with profound metabolic and vascular alterations and is believed to play an important role in initiating migraine attacks [6,19].

The initiation and propagation of CSD impose a substantial energetic burden on cortical tissue [19]. During the early phase of CSD, intense neuronal activity leads to rapid consumption of cellular energy substrates and reducing equivalents such as NADH. This increased metabolic demand may transiently exceed oxygen availability, resulting in a state of functional hypoxia and enhanced mitochondrial production of ROS [19,20].

Experimental studies have demonstrated that CSD is associated with increased oxidative stress in brain tissue. In animal models, CSD has been shown to elevate extracellular hydrogen peroxide (H_2_O_2_) levels by approximately 20% and to increase concentrations of malondialdehyde (MDA), a marker of lipid peroxidation, by nearly 67% in the cerebral cortex. In meningeal tissues, MDA levels may increase by approximately 70%, indicating significant oxidative damage within structures involved in migraine pain generation [20].

These findings suggest that oxidative stress generated during CSD may represent an important link between metabolic dysfunction and the activation of downstream nociceptive pathways involved in migraine pathophysiology [20].

**Figure 2:**
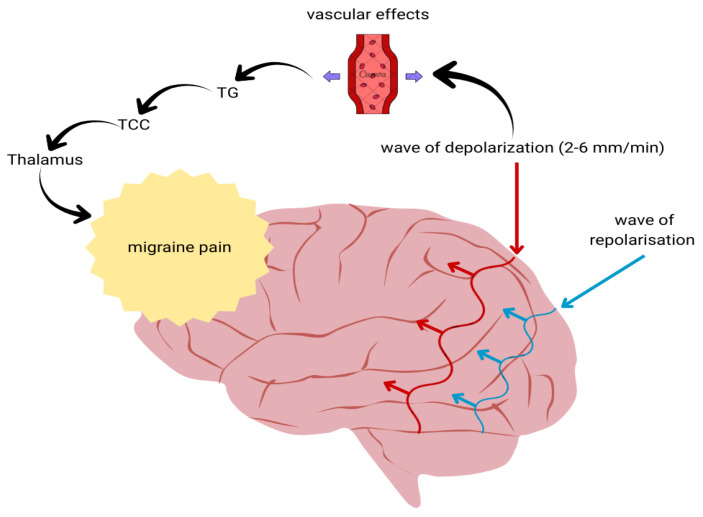
Cortical spreading depression and its role in trigeminovascular activation in migraine. CSD represents a slowly propagating wave of neuronal depolarization followed by repolarization that travels across the cerebral cortex at a rate of approximately 2–6 mm/min. CSD induces vascular changes and activates trigeminal nociceptive pathways involving the TG, TCC, and thalamus, ultimately contributing to migraine pain. Purple arrows denote the bidirectional neurovascular interplay associated with cortical spreading depolarization. CSD—cortical spreading depression; TG—trigeminal ganglion; TCC—trigeminocervical complex.

The main mechanisms involved in CSD propagation and its relationship with trigeminal nociceptive pathways are illustrated in Figure 2.

### 2.4. TRPA1 Activation, Migraine Triggers, and Trigeminovascular Signaling

Transient receptor potential ankyrin 1 (TRPA1) channels are non-selective cation channels expressed predominantly in nociceptive sensory neurons, including trigeminal afferents involved in migraine pain transmission. These channels can be activated by reactive oxygen and nitrogen species as well as by electrophilic compounds generated during oxidative stress, linking redox imbalance to nociceptive signaling pathways involved in migraine pathophysiology [16,21,22].

Experimental evidence suggests that TRPA1 channel activation may play an important role in the trigeminovascular system, a key pathway in migraine pathogenesis. In experimental models, stimulation of TRPA1 channels located on trigeminal nerve endings has been shown to promote neuronal depolarization and the release of CGRP and other vasoactive mediators. CGRP is a potent vasodilator and a major mediator of neurogenic inflammation, and it is considered to contribute to meningeal vasodilation and sensitization of nociceptive pathways during migraine attacks [16,23].

A variety of environmental, dietary, and physiological factors known to trigger migraine attacks may act through mechanisms that increase oxidative stress and subsequently activate TRPA1 channels. These triggers include dietary factors such as alcohol, monosodium glutamate, aspartame, tyramine, phenylethylamine, and nitrates; environmental stimuli such as noise, pollution, and weather changes; physiological conditions including hypoglycemia, hypoxia, infection, and hormonal fluctuations; as well as behavioral factors such as sleep disturbances, psychological stress, and mental overexertion [20,21].

Several molecular mechanisms may contribute to the generation of oxidative stress in response to these triggers. These include increased mitochondrial activity during high metabolic demand, mitochondrial toxicity or membrane alterations, calcium overload and excitotoxicity, activation of NADPH oxidases in neurons, neuroinflammatory responses involving microglial activation, and the generation of reactive species as by-products of monoamine oxidase or cytochrome P450 metabolism. Additionally, uncoupling of nitric oxide synthase may further enhance the production of reactive oxygen species [20,21,24].

Because many migraine triggers share the ability to increase oxidative stress and activate TRPA1 channels, this pathway may represent a potential mechanistic link between environmental stimuli, oxidative stress–induced TRPA1 activation, and CGRP-mediated trigeminovascular responses in migraine pathophysiology [20,21,22]. However, much of the available evidence regarding TRPA1 signaling in migraine derives from preclinical and experimental models, and further clinical studies are needed to clarify its relevance in human migraine.

### 2.5. Therapeutic Implications of Targeting Oxidative Stress

The pathophysiological mechanisms discussed above suggest that oxidative stress, mitochondrial dysfunction, and trigeminovascular activation represent potential therapeutic targets in migraine. Several treatment strategies aim to modulate these pathways by improving mitochondrial function, reducing oxidative stress, or interfering with neurogenic inflammation. These approaches include nutraceutical supplementation, pharmacological therapies, and emerging targeted treatments [19,20].

#### 2.5.1. Nutraceutical and Metabolic Interventions

Several nutraceutical compounds have been investigated for migraine prevention due to their ability to improve mitochondrial function and reduce oxidative stress. Riboflavin (vitamin B2) has demonstrated efficacy in several clinical trials and is thought to enhance mitochondrial energy metabolism while supporting the recycling of oxidized glutathione, thereby reducing oxidative stress [25,26,27,28].

Other compounds with potential benefits include coenzyme Q10 and alpha-lipoic acid, both known for their antioxidant properties and their role in mitochondrial bioenergetics [19,20]. B-complex vitamins such as thiamine, folate, niacin, and cobalamin may also contribute to improved mitochondrial metabolism. Magnesium supplementation has received particular attention, as magnesium deficiency has frequently been reported in migraine patients. Magnesium may inhibit cortical spreading depression by blocking calcium channels in neurons and reducing neuronal hyperexcitability [15,19].

L-carnitine has also been explored as a potential therapeutic option due to its role in transporting fatty acids into mitochondria for β-oxidation and energy production [19].

Metabolic interventions such as ketogenic diets have been proposed as alternative therapeutic strategies. Ketosis enables the brain to utilize ketone bodies as an alternative energy substrate, potentially improving mitochondrial efficiency and reducing neuronal excitability. Ketogenic metabolism may also enhance antioxidant defenses, increase the expression of glucose and ketone transporters, and promote inhibitory neurotransmission through increased γ-aminobutyric acid (GABA) signaling [19,20]. In addition, aerobic exercise may contribute to improved mitochondrial efficiency and cerebral energy metabolism through adaptive metabolic responses [19].

#### 2.5.2. Pharmacological Therapies

Topiramate has been shown to enhance inhibitory neurotransmission and reduce the release of excitatory neurotransmitters such as glutamate and CGRP. Although topiramate may exert neuroprotective effects in certain contexts, its relationship with oxidative stress appears complex and may vary depending on dose and cellular conditions [23,29].

Amitriptyline has been associated with reduced oxidative stress markers and increased antioxidant capacity, while valproate may exert beneficial effects on mitochondrial function and has been shown to stimulate mitochondrial biogenesis [26]. In addition, beta-blockers remain widely used in migraine prevention and may reduce metabolic demand and sympathetic activation, potentially influencing oxidative stress pathways indirectly [19,23].

Abortive therapies may also influence metabolic pathways. For example, corticosteroids and caffeine have been associated with stimulation of gluconeogenesis and modulation of neuronal excitability, while caffeine may additionally inhibit TRPA1 channel activity [19].

#### 2.5.3. Emerging Targeted Therapies

Recent advances in migraine therapy include monoclonal antibodies targeting CGRP or its receptor. Currently available agents include eptinezumab, fremanezumab, galcanezumab, and erenumab. These therapies have demonstrated significant efficacy in reducing migraine frequency and are generally well tolerated, particularly in patients who are refractory to conventional preventive treatments [30,31].

In addition to CGRP-targeted therapies, TRPA1 channels have emerged as potential therapeutic targets due to their role in linking oxidative stress to trigeminovascular activation. Experimental TRPA1 antagonists, such as GRC17536, are currently being investigated and may represent promising future therapeutic options for migraine management [16,22].

Therefore, therapeutic strategies targeting mitochondrial dysfunction, oxidative stress, and trigeminovascular signaling pathways may represent promising approaches for migraine prevention and treatment.

However, several important questions remain unresolved. Although CGRP-targeted therapies have shown substantial clinical benefit, their long-term effects on vascular and neuroinflammatory pathways, as well as their differential efficacy across migraine subtypes, still require further investigation. Likewise, TRPA1-targeted approaches remain at an early stage of development, and their translational relevance for routine clinical practice has yet to be clearly established.

Beyond neuronal and glial mechanisms, cortical spreading depression also induces profound alterations in cerebral vascular tone and endothelial function [32]. These neurovascular interactions are closely linked to nitric oxide signaling pathways, which play a key role in regulating cerebrovascular responses during migraine attacks.

## 3. Nitric Oxide–cGMP Signaling and Endothelial Dysfunction in Migraine

One of the key downstream pathways of NO signaling involves activation of soluble guanylate cyclase (sGC) and subsequent production of cyclic guanosine monophosphate (cGMP). The NO–sGC–cGMP cascade plays a central role in vascular smooth muscle relaxation and regulation of cerebral vascular tone [32]. Dysregulation of this pathway has been proposed as a potential mechanism contributing to abnormal neurovascular responses observed in migraine patients, particularly during headache attacks [32,33].

**Figure 3:**
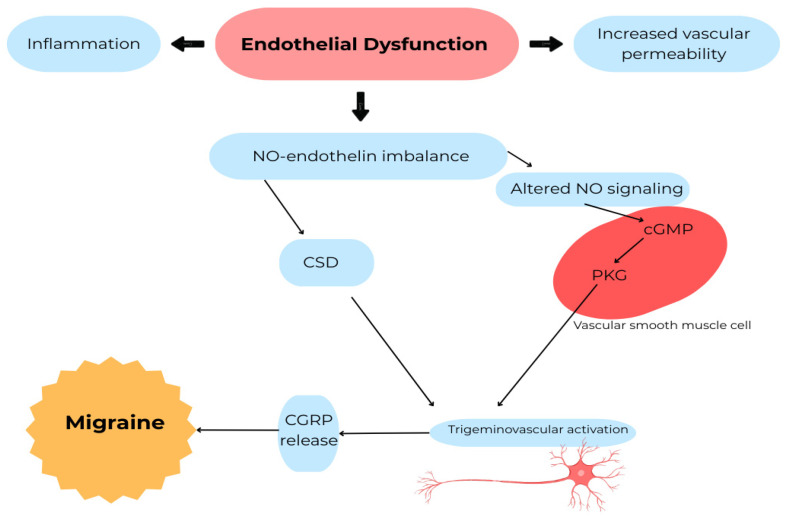
Endothelial dysfunction in migraine pathophysiology. Endothelial dysfunction promotes inflammatory responses and increased vascular permeability with an imbalance between nitric oxide and endothelin signaling. Altered nitric oxide signaling activates the cGMP–PKG pathway in vascular smooth muscle cells, contributing to trigeminovascular activation and CGRP release. These processes interact with CSD and neurovascular signaling pathways involved in migraine development. CGRP—calcitonin gene-related peptide; CSD—cortical spreading depression; NO—nitric oxide; cGMP—cyclic guanosine monophosphate; PKG—protein kinase G.

Experimental evidence supporting the involvement of NO in migraine pathophysiology has been obtained from studies using nitric oxide donors. Administration of nitroglycerin or other NO-releasing compounds can reliably induce delayed migraine-like attacks in susceptible individuals and has therefore been widely used as an experimental model for investigating migraine mechanisms and evaluating potential therapeutic interventions [34]. The delayed onset of headache following nitroglycerin administration suggests that nitric oxide may trigger a cascade of downstream molecular events involved in migraine initiation [34]. However, the extent to which nitroglycerin-induced headache fully reproduces the complexity of spontaneous migraine attacks remains uncertain, and this model should be interpreted primarily as a useful experimental tool rather than a complete representation of clinical migraine. The main mechanisms linking endothelial dysfunction, nitric oxide signaling, and trigeminovascular activation in migraine are summarized in Figure 3.

Another mechanism linking nitric oxide signaling to migraine involves NOS uncoupling. Under conditions of oxidative stress or reduced availability of essential cofactors such as tetrahydrobiopterin (BH4), NOS enzymes may become uncoupled and produce superoxide rather than nitric oxide [35]. This process leads to reduced NO bioavailability and increased oxidative stress, contributing to endothelial dysfunction and impaired vascular regulation. Such alterations may play an important role in migraine-associated vascular dysregulation [35].

Clinical studies further support the involvement of nitric oxide-related pathways in migraine. Patients with migraine, particularly those with migraine with aura, have been shown to exhibit impaired endothelial function and altered vascular reactivity [36]. Reduced nitric oxide bioavailability and increased oxidative stress may therefore contribute to cerebrovascular dysregulation and may partially explain the association between migraine and increased risk of cerebrovascular events reported in epidemiological studies [36,37].

## 4. Endothelial Dysfunction and Vascular Alterations in Migraine

The vascular endothelium plays a crucial role in maintaining cerebrovascular homeostasis through the regulation of vascular tone, inflammatory responses, and hemostatic balance [37]. Endothelial cells release several vasoactive mediators that regulate vascular reactivity and cerebral blood flow, and disruption of these mechanisms may lead to abnormal neurovascular responses. Increasing evidence suggests that migraine is associated with systemic vascular alterations, indicating that endothelial dysfunction may contribute to the broader pathophysiological mechanisms underlying the disorder [37,38].

Clinical investigations have demonstrated impaired endothelial function in individuals with migraine [37]. Studies evaluating endothelial-dependent vasodilation using techniques such as flow-mediated dilation have reported reduced vascular reactivity in migraine populations compared to healthy controls. In addition, several circulating biomarkers associated with endothelial activation and vascular dysfunction—including markers related to oxidative stress, endothelial injury, and inflammation—have been reported to differ between migraine patients and non-migraine controls [39]. These findings support the concept that migraine may involve systemic vascular dysregulation rather than representing a purely neuronal disorder.

Endothelial dysfunction may also contribute to the increased cerebrovascular risk observed in migraine populations. Epidemiological studies have consistently shown that migraine, particularly migraine with aura, is associated with a higher risk of ischemic stroke and other vascular events [40]. Several mechanisms have been proposed to explain this association, including endothelial activation, impaired vascular reactivity, platelet aggregation, and chronic vascular inflammation [41]. Although the causal pathways remain incompletely understood, endothelial dysfunction is increasingly considered an important mechanistic link between migraine and vascular disease.

Nevertheless, the available evidence remains heterogeneous, and it is still unclear whether endothelial dysfunction represents a primary pathophysiological driver, a secondary phenomenon, or a context-dependent marker of increased vascular susceptibility in migraine.

## 5. Neuroinflammation in Migraine

Growing evidence indicates that neuroinflammatory mechanisms play an important role in migraine pathophysiology [34,42]. Beyond neuronal and vascular alterations, migraine attacks appear to involve complex interactions between the nervous system and immune signaling pathways [42]. Activation of inflammatory processes within the trigeminovascular system and surrounding meningeal tissues may contribute to the initiation and amplification of migraine pain. Several studies suggest that inflammatory mediators released in these regions can influence neuronal excitability and facilitate nociceptive signaling, highlighting the importance of immune–neural interactions in migraine mechanisms [42].

Experimental studies have shown that activation of glial cells, particularly microglia and astrocytes, may contribute to the amplification of nociceptive signaling during migraine attacks [42,43]. Activated microglia release a range of pro-inflammatory cytokines, including interleukin-1β (IL-1β), tumor necrosis factor-α (TNF-α), and interleukin-6 (IL-6), which can increase neuronal excitability and promote central sensitization [43]. In experimental migraine models, microglial activation has been associated with enhanced inflammatory signaling through pathways such as nuclear factor-κB (NF-κB), further supporting the role of neuroimmune mechanisms in migraine pathogenesis [43].

**Figure 4:**
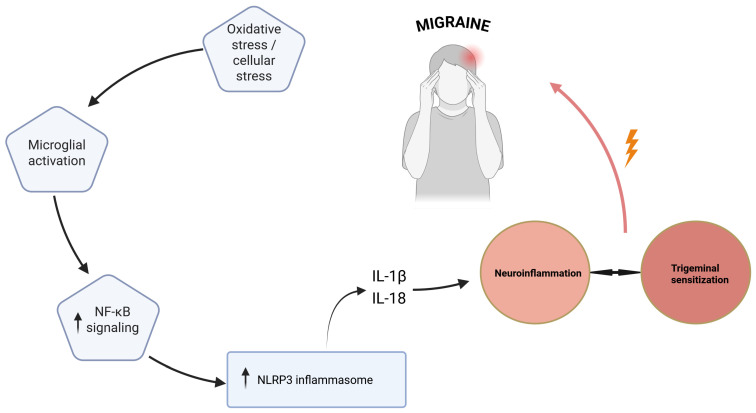
Putative neuroinflammatory mechanisms involving NLRP3 inflammasome signaling in migraine. Oxidative stress and cellular stress may promote microglial activation and NF-κB signaling, leading to increased NLRP3 inflammasome activation and the release of pro-inflammatory cytokines such as IL-1β and IL-18. These processes may contribute to neuroinflammation, trigeminal sensitization, and migraine pain, potentially forming a self-amplifying cycle. Upward arrows indicate increased activation or expression. NF-κB—nuclear factor kappa B; NLRP3—NOD-, LRR- and pyrin domain-containing protein 3; IL—interleukin.

Another proposed mechanism linking inflammation and migraine involves activation of the NLRP3 inflammasome, a cytosolic protein complex responsible for the maturation and release of inflammatory cytokines such as IL-1β and IL-18 [44]. Activation of this pathway has been observed primarily in experimental models of migraine and may contribute to inflammatory signaling within trigeminal pathways. In addition, immune cells located in meningeal tissues can release cytokines and other inflammatory mediators capable of sensitizing trigeminal afferents, thereby contributing to the persistence and amplification of migraine pain [43,44]. The putative neuroinflammatory mechanisms linking oxidative stress, microglial activation, NLRP3 inflammasome signaling, and trigeminal sensitization in migraine are illustrated in Figure 4. However, most of the available evidence regarding NLRP3 inflammasome activation in migraine is derived from preclinical studies, and its precise role in human migraine remains to be fully elucidated. Together, these findings suggest that neuroinflammation represents an important component of migraine pathophysiology and may provide potential targets for future therapeutic strategies.

## 6. Clinical Implications and Comorbidities Associated with Migraine

Migraine is increasingly recognized not only as a primary neurological disorder but also as a systemic condition frequently associated with multiple comorbid diseases. Large epidemiological studies and comprehensive reviews have highlighted that migraine commonly coexists with several neurological, autoimmune, and chronic pain disorders, suggesting the involvement of shared biological pathways [3,45]. Mechanisms such as oxidative stress, chronic low-grade inflammation, mitochondrial dysfunction, and endothelial impairment—discussed in the previous sections—may contribute both to migraine pathogenesis and to the development of these comorbid conditions. Among the disorders most frequently investigated in relation to migraine are Parkinson’s disease, fibromyalgia, and Sjögren’s syndrome, although the precise nature of these associations remains incompletely understood [3,45].

### 6.1. Migraine and Parkinson’s Disease

Migraine and PD are chronic neurological disorders with distinct clinical manifestations and therapeutic approaches, yet growing evidence suggests potential links between the two conditions. Several epidemiological studies indicate that migraine, particularly migraine with aura, may be associated with an increased risk of developing PD later in life [46,47]. The magnitude of this risk appears to be influenced by factors such as age, sex, pre-existing comorbidities, and socioeconomic status [48]. One of the most frequently proposed biological connections involves the dopaminergic system, which plays a role in both migraine pathophysiology and the neurodegenerative processes underlying Parkinson’s disease [49].

Evidence supporting this association comes from a long-term cohort study that followed 5620 individuals aged 33–65 years over a 25-year period. At baseline, participants were categorized as having no headaches, non-migraine headaches, migraine without aura, or migraine with aura. During follow-up, individuals with migraine with aura were more than twice as likely to develop PD compared with those without headaches (2.4% vs. 1.1%) [50]. They also had significantly higher odds of presenting multiple parkinsonian symptoms, with nearly 20% of participants with migraine with aura reporting such manifestations compared with lower proportions in individuals with migraine without aura or no headache history. Additionally, women with migraine with aura more frequently reported a family history of PD, further suggesting a possible shared biological susceptibility [50].

Several mechanisms have been proposed to explain this relationship. Dopamine dysregulation represents one of the most plausible links, as migraine has been associated with dopaminergic hypersensitivity, whereas PD is characterized by progressive dopaminergic neuron loss in the substantia nigra [46]. This shared involvement of dopaminergic pathways may contribute to overlapping clinical manifestations, including symptoms such as yawning, nausea, and vomiting that are partly mediated by dopamine receptor activity [50]. In addition, both conditions have been associated with mitochondrial dysfunction, neuroinflammation, and increased oxidative stress, suggesting that common molecular pathways may contribute to their coexistence. Clinically, migraine symptoms may sometimes improve as PD progresses, possibly due to degeneration of dopaminergic neurons that reduces dopaminergic hypersensitivity; however, studies have not consistently demonstrated a direct correlation between migraine improvement and the onset of motor features of PD [51]. Management can also be challenging, as certain dopaminergic medications may exacerbate migraine in susceptible patients, while standard migraine therapies must be used cautiously due to potential drug interactions or adverse effects in older patients with PD [52,53].

### 6.2. Migraine and Fibromyalgia

Fibromyalgia is a chronic pain disorder characterized by widespread musculoskeletal pain accompanied by fatigue, sleep disturbances, and cognitive symptoms. Although its pathophysiology remains incompletely understood, current evidence suggests a multifactorial origin involving genetic susceptibility, neuroendocrine alterations, inflammation, and stress-related mechanisms [54,55]. Endocrine abnormalities, including dysregulation of the hypothalamic–pituitary–adrenal and hypothalamic–pituitary–thyroid axes, have also been reported, with altered levels of cortisol, growth hormone, insulin-like growth factor, and thyroid-related hormones observed in some patients [56]. These neuroendocrine and inflammatory disturbances may partially overlap with mechanisms implicated in migraine, particularly those involving hypothalamic regulation and neuropeptide signaling [57].

A key mechanism in fibromyalgia pathogenesis is central sensitization, characterized by increased responsiveness of nociceptive neurons within the central nervous system and amplification of pain perception [58]. This phenomenon involves enhanced synaptic transmission mediated by excitatory neurotransmitters such as glutamate acting through NMDA and AMPA receptors, leading to increased neuronal excitability and the “wind-up” phenomenon in spinal cord neurons [59,60]. Dysregulation of pain-modulating neurotransmitters—including serotonin and substance P—has also been described in both fibromyalgia and migraine, suggesting overlapping neurochemical pathways that may contribute to pain facilitation in these disorders [61,62,63,64].

The frequent coexistence of migraine and fibromyalgia suggests shared neurobiological mechanisms involving central sensitization and altered pain processing, including central sensitization, neuroinflammation, and altered pain processing networks. Glial activation, increased pro-inflammatory cytokine signaling, and dysfunction of descending inhibitory pain pathways may contribute to persistent pain amplification in both conditions [54,56]. Neuroimaging studies further support this overlap, demonstrating altered brain connectivity and abnormal pain processing in regions involved in sensory integration and CSD [65,66]. These shared mechanisms—including enhanced neural excitability, neuroplastic changes, and neuroinflammatory processes—may explain the frequent comorbidity of migraine and fibromyalgia and suggest that therapeutic strategies targeting these pathways could benefit both conditions [58,67,68,69,70,71,72,73].

### 6.3. Migraine and Sjögren’s Disease

Sjögren’s disease (SD) and syndrome are two distinct conditions, but they can occur together, and there are connections between them, particularly in how autoimmune processes and neurological symptoms overlap. SD is a chronic autoimmune disorder where the immune system targets the exocrine glands (especially the salivary and lacrimal glands), leading to xerostomia and xerophthalmia [74]. Systemic symptoms, including fatigability, arthralgia, peripheral neuropathy, and central nervous system dysfunction, can be associated with it. Headaches, including migraine-like headaches, are reported more frequently than in the general population. SD-related vasculitis, cerebral small vessel disease, or inflammation in the CNS could potentially trigger or worsen migraines [75]. However, the direction of this association remains unclear, as migraine-like headache may reflect immune-mediated neuroinflammation, vascular dysfunction, or central sensitization related to SD rather than a disease-specific manifestation.

The pain threshold for migraine may be lowered by increased proinflammatory cytokines by chronic inflammation associated with SD. Proinflammatory cytokines contribute to central sensitization by activating the trigeminovascular system. Some therapies used in SD, including corticosteroids and immunosuppressive agents, may also indirectly influence migraine frequency and symptom burden [76].

Taken together, these associations highlight the complex systemic dimension of migraine and suggest that several comorbid conditions may share overlapping biological pathways. Mechanisms such as oxidative stress, mitochondrial dysfunction, neuroinflammation, endothelial impairment, and central sensitization represent common elements linking migraine with other neurological, autoimmune, and chronic pain disorders. These interconnected molecular and neurovascular processes may contribute not only to disease coexistence but also to increased clinical burden and therapeutic challenges. Recognizing these shared mechanisms may facilitate a more integrated understanding of migraine pathophysiology and open new perspectives for therapeutic strategies targeting common pathogenic pathways.

## 7. Future Perspectives and Therapeutic Implications

Recent advances in migraine research have highlighted the complex interactions between oxidative stress, neurovascular signaling, and inflammatory pathways involved in migraine pathophysiology. However, many aspects of these mechanisms remain incompletely understood, particularly regarding the interactions between redox imbalance, endothelial dysfunction, and neuroimmune activation. Future studies should therefore focus on identifying reliable molecular biomarkers associated with oxidative stress and neuroinflammation that could improve diagnosis and therapeutic stratification of migraine patients [32]. In addition, emerging therapeutic approaches targeting inflammatory signaling pathways, mitochondrial dysfunction, and oxidative stress may represent promising directions for the development of novel migraine treatments [77,78].

As a narrative review, the present manuscript is limited by its non-systematic design and by the heterogeneity of the available clinical and preclinical evidence, which may affect the generalizability of some of the discussed mechanisms and therapeutic perspectives.

## 8. Conclusions

Migraine represents a complex neurovascular disorder in which oxidative stress, nitric oxide signaling, endothelial dysfunction, and neuroinflammatory mechanisms interact to drive neuronal sensitization and abnormal vascular responses. Current evidence suggests that these processes interplay in a pathophysiological network linking redox imbalance, vascular dysregulation, and immune activation within trigeminovascular pathways. Understanding these interactions provides important insights into migraine biology and highlights potential molecular targets for therapeutic intervention, although several of these mechanisms still require further validation in human studies. Future research integrating molecular, vascular, and clinical approaches will be essential for identifying reliable biomarkers and developing more targeted treatment strategies for migraine.

## Data Availability

No new data were created or analyzed in this study. Data sharing is not applicable to this article.

## Abbreviations

The following abbreviations are used in this manuscript:

AMPA	α-Amino-3-Hydroxy-5-Methyl-4-Isoxazolepropionic Acid Receptor
BH4	Tetrahydrobiopterin
CGRP	Calcitonin Gene-Related Peptide
CSD	Cortical Spreading Depression
cGMP	Cyclic Guanosine Monophosphate
eNOS	Endothelial Nitric Oxide Synthase
GABA	Gamma-Aminobutyric Acid
IL-1β	Interleukin-1 Beta
IL-6	Interleukin-6
iNOS	Inducible Nitric Oxide Synthase
MDA	Malondialdehyde
MRS	Magnetic Resonance Spectroscopy
nNOS	Neuronal Nitric Oxide Synthase
NADH	Nicotinamide Adenine Dinucleotide (Reduced Form)
NF-κB	Nuclear Factor Kappa B
NMDA	N-Methyl-D-Aspartate
NO	Nitric Oxide
NOS	Nitric Oxide Synthase
NOX	NADPH Oxidase
NLRP3	NOD-, LRR- and Pyrin Domain-Containing Protein 3
PD	Parkinson’s Disease
PKG	Protein Kinase G
RNS	Reactive Nitrogen Species
ROS	Reactive Oxygen Species
SD	Sjögren Disease
sGC	Soluble Guanylate Cyclase
TCC	Trigeminocervical Complex
TG	Trigeminal Ganglion
TNF-α	Tumor Necrosis Factor Alpha
TRPA1	Transient Receptor Potential Ankyrin 1
